# Influence of Organic Impurities on Fractional Crystallization of NaCl and Na_2_SO_4_ from High-Salinity Coal Chemical Wastewater: Thermodynamics and Nucleation Kinetics Analysis

**DOI:** 10.3390/molecules29091928

**Published:** 2024-04-23

**Authors:** Bo Shen, Bo Zhao, Hai Du, Yongsheng Ren, Jianwei Tang, Yong Liu, Quanxian Hua, Baoming Wang

**Affiliations:** 1 School of Ecology and Environment, Zhengzhou University, Zhengzhou 450001, China; 2 National Center for Research & Popularization on Calcium, Magnesium, Phosphate and Compound Fertilizer Technology, Zhengzhou 450001, China; 3 Research Centre of Engineering and Technology for Synergetic Control of Environmental Pollution and Carbon Emissions of Henan Province, Zhengzhou 450001, China; 4 School of Chemical Engineering, Zhengzhou University, Zhengzhou 450001, China; 5 State Key Laboratory of High-Efficiency Utilization of Coal and Green Chemical Engineering, Ningxia University, Yinchuan 750021, China

**Keywords:** high-salinity wastewater, thermodynamics, phase equilibrium, crystallization kinetics

## Abstract

It is a valid path to realize the zero discharge of coal chemical wastewater by using the fractional crystallization method to recycle the miscellaneous salt in high-salinity wastewater. In this study, the thermodynamics and nucleation kinetics of sodium chloride (NaCl) and sodium sulfate (Na_2_SO_4_) crystallization in coal chemical wastewater were systematically studied. Through analyses of solubility, metastable zone width, and induction period, it was found that the impurity dimethoxymethane would increase the solid–liquid interface energy and critical crystal size during the nucleation of Na_2_SO_4_. Ternary phase diagrams of the pseudo-ternary Na_2_SO_4_-NaCl-H_2_O systems in simulated wastewater were plotted in the temperature range of 303.15 to 333.15 K, indicating that a co-ionization effect existed between NaCl and Na_2_SO_4_, and NaCl had a strong salting out effect on Na_2_SO_4_. Finally, the nucleation rate and growth rate of Na_2_SO_4_ crystals under simulated wastewater conditions were determined by the intermittent dynamic method, and the crystallization kinetic models of Na_2_SO_4_ were established. The crystallization nucleation of Na_2_SO_4_ crystals was found to be secondary nucleation controlled by surface reactions. The basic theoretical research of crystallization in this study is expected to fundamentally promote the application of fractional crystallization to realize the resource utilization of high-salinity wastewater in the coal chemical industry.

## 1. Introduction

The contradiction between the modern coal chemical industry and the requirements of saving resources and protecting the environment is becoming increasingly prominent [1,2]. Large amounts of high-salinity wastewater and organic wastewater are produced during the modern coal chemical production process, usually containing various soluble inorganic salts, such as Cl^−^, Na^+^, and SO_4_^2−^, etc. The discharge of such wastewater inevitably leads to the mineralization of freshwater resources and soil alkalization [3]. The effective treatment and separation of miscellaneous salt resources are of great significance for improving the utilization value of high-salinity wastewater in the coal chemical industry and achieving zero discharge [4,5].

High-salinity wastewater from the coal chemical industry is characterized by high contents of inorganic salt ions, a poor biodegradability, and a complex composition [6]. Generally, wastewater with salinity in the range of 1 to 3.5% *w*/*w* salts is termed as highly saline, while that containing more than 3.5% *w*/*w* salts present in oceans is termed hypersaline [7]. The treatment methods for such wastewater in China mainly include evaporation crystallization and evaporation pond salt drying [8]. However, both methods produce mixed crystalline salts with high contents of heavy metals and organic matter, which are complex in composition and difficult to use, resulting in a low degree of resource utilization [9]. In this context, the zero-discharge technological requirement with the core concept of fractional crystallization was proposed, which not only concerns the water treatment, but also focuses more on the recycling of the dissolved inorganics [10].

The theory of fractional crystallization is of great significance for guiding its practical application, mainly including crystallization thermodynamics and nucleation kinetic properties. It is known that typical coal chemical wastewater is rich in sodium sulfate (Na_2_SO_4_) and sodium chloride (NaCl), which is considered as a classical water-salt system. To date, the crystallization processes of ternary, quaternary, and pluralistic systems containing Na_2_SO_4_ or NaCl have been extensively studied, involving phase equilibrium [11,12,13], crystallization kinetics [14], and process optimization [15]. Zhang et al. [16] investigated the solid-liquid equilibrium for the ternary Na_2_SO_4_-NaCl-H_2_O system at 313.15 K. Zeng et al. [17] estimated the primary nucleation kinetics during Na_2_SO_4_ crystallization at temperatures ranging from 313.15 to 353.15 K. Bian et al. [15] conducted process parameters optimization on Na_2_SO_4_ fractional crystallization in a quaternary NaCl-NaNO_3_-Na_2_SO_4_-H_2_O system. Despite a lot of published studies, only some of crystallization fundamentals have been involved, either thermodynamics or nucleation kinetics, and few studies have been reported that cover both aspects. Compared to thermodynamics, fewer studies on nucleation kinetics have been published, even in the classical Na_2_SO_4_-NaCl-H_2_O system. In this study, thermodynamic properties, phase equilibrium regularity, and nucleation kinetics were comprehensively investigated in a ternary Na_2_SO_4_-NaCl-H_2_O system covering temperatures ranging from 278.15 to 303.15 K.

The existence of non-negligible organic impurities in wastewater will inevitably influence the crystallization process and the quality of subsequent salts [18]. Previous studies have shown that additives have influences on the thermodynamics, kinetics, and morphology of salt crystallization [19,20,21]. Zhu et al. [22] plotted the phase diagrams of NaCl and Na_2_SO_4_ in the presence of cyclohexanol and cyclohexanone and obtained different influence trends. Rajesh studied the effect of EDTA on the metastable zone of ammonium dihydrogen phosphate (ADP) [23], indicating that EDTA significantly increased the metastable zone width of ADP and decreased the nucleation rate. The above reports not only prove that organic impurities affect the crystallization of salts more or less, but also imply that the corresponding research in the water-salt system cannot be generalized. Wastewater composition varies greatly, and fundamental crystallization research on different wastewater systems is necessary to supplement the high-salinity wastewater system theory [14]. The influences of different organic impurities on solubility, metastable zone width, induction period, and nucleation kinetics should be further studied [24].

Based on the above considerations, targeting high-salinity wastewater from the coal chemical industry in Ningxia, for the first time, this study theoretically revealed the influence of dimethoxymethane on the crystallization thermodynamic properties and crystal nucleation process of Na_2_SO_4_ and NaCl. According to the analysis report, the main inorganic salts involved in the Nixia wastewater were Na_2_SO_4_ and NaCl, with content ranges of 1.2 to 2.0% and 2.3 to 3.0% *w*/*w*, respectively. The most abundant organic compositions included dimethoxymethane, diethyl ether, 1,1 dichloroethane, and dibromomethane et al., among which, dimethoxymethane ranked the first. Therefore, in the present study, dimethoxymethane was selected as a typical organic impurity in a classical Na_2_SO_4_-NaCl-H_2_O system to conduct the research. It is expected that the results could provide theoretical guidance for the separation and resource utilization of Na_2_SO_4_ and NaCl.

## 2. Results and Discussion

### 2.1. Effect of Dimethoxymethane on Thermodynamics Properties of NaCl and Na_2_SO_4_ Crystallization

#### 2.1.1. Solubility of Na_2_SO_4_ and NaCl

**Figure 1:**
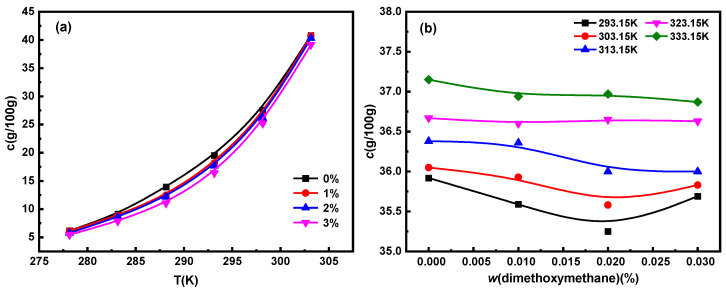
Solubility of (**a**) Na_2_SO_4_ and (**b**) NaCl at different temperatures and dimethoxymethane concentrations.

The solubility of Na_2_SO_4_ and NaCl in water at different temperatures and dimethoxymethane concentrations in the temperature range of 278.15 to 303.15 K are shown in Figure 1. The solubility of Na_2_SO_4_ and NaCl increase with a rise in temperature. The solubility data of Na_2_SO_4_ were linearly fitted by the Apelblat equation, Van ‘t Hoff equation, and polynomial equation, respectively, among which, the Van ‘t Hoff model was the most suitable (Table S1). Based on the Van ‘t Hoff model, the Gibbs free energy of the dissolution of Na_2_SO_4_ in dimethoxymethane solution at different concentrations was estimated (Table S2). All the dissolution enthalpy and Gibbs free energy values were positive, indicating that the dissolution of Na_2_SO_4_ in dimethoxymethane solutions was an endothermic reaction. The solubility of Na_2_SO_4_ increased with an increase in temperature, which was consistent with previous experimental results [25]. At the same time, all the dissolution enthalpy values were greater than those of the dissolution entropy, indicating that the main driving force of Na_2_SO_4_ in the dissolution process was the dissolution enthalpy.

At the same temperature, with an increase in dimethoxymethane concentration, the solubility of Na_2_SO_4_ decreased significantly, while that of NaCl first decreased and then increased. As a whole, dimethoxymethane inhibited the dissolution of Na_2_SO_4_ and NaCl. When Na_2_SO_4_ and NaCl were dissolved in water, Cl^−^, SO_4_^2−^, and Na^+^ were ionized, forming three unstable hydrated ions by binding water molecules through intermolecular forces and electrostatic interactions [26]. The ether group –O– in the structure of dimethoxymethane is a hydrophilic group [27], which could have competed with hydrated ions for water molecules in the solvent, thereby reducing the solubility of Na_2_SO_4_ and NaCl. The slightly increased solubility of NaCl at 3% dimethoxymethane might have been caused by the interaction between NaCl and Na_2_SO_4_.

#### 2.1.2. The Metastable Zone Width of Na_2_SO_4_ Solution

**Figure 2:**
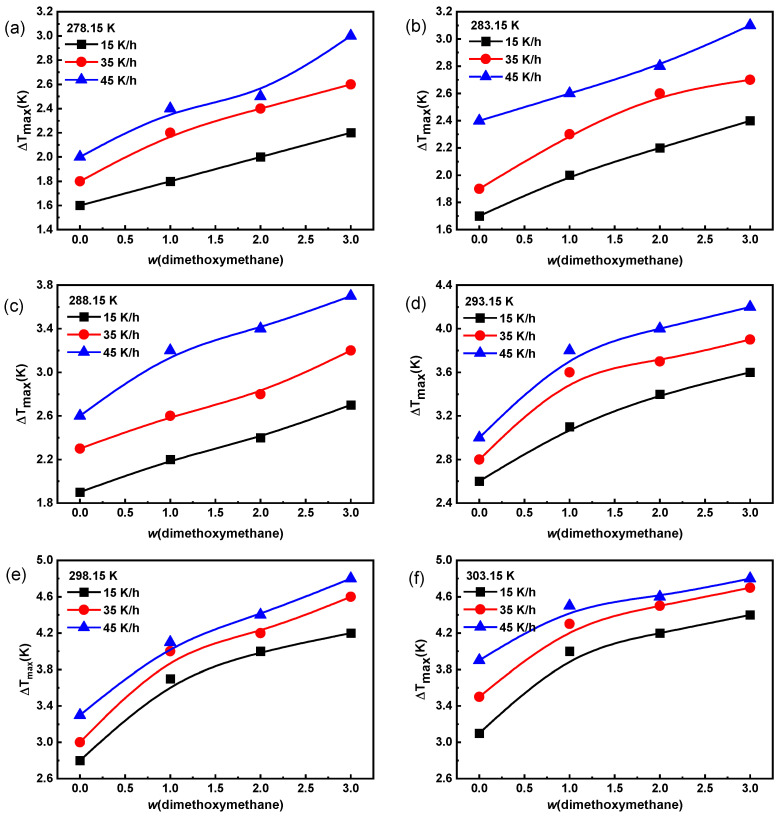
The effect of dimethoxymethane on the metastable zone width of Na_2_SO_4_ in water with different cooling rates at saturation temperatures: (**a**) 278.15 K; (**b**) 283.15 K; (**c**) 288.15 K; (**d**) 293.15 K; (**e**) 298.15 K; and (**f**) 303.15 K.

The metastable zone width is an important parameter of crystallization thermodynamics [17]. The effects of temperature, dimethoxymethane concentration, and cooling rate on the metastable zone width of Na_2_SO_4_ are shown in Figure 2. The metastable zone width of Na_2_SO_4_ solution widened with an increase in cooling rate, saturation temperature, and dimethoxymethane concentration, making it difficult for Na_2_SO_4_ to crystallize in water. As the cooling rate increased, the time for the solute to pass through the nucleation temperature zone was too short for the crystals to precipitate in time, resulting in a lag in crystal precipitation and, thus, a widening of the metastable zone [28].

**Figure 3:**
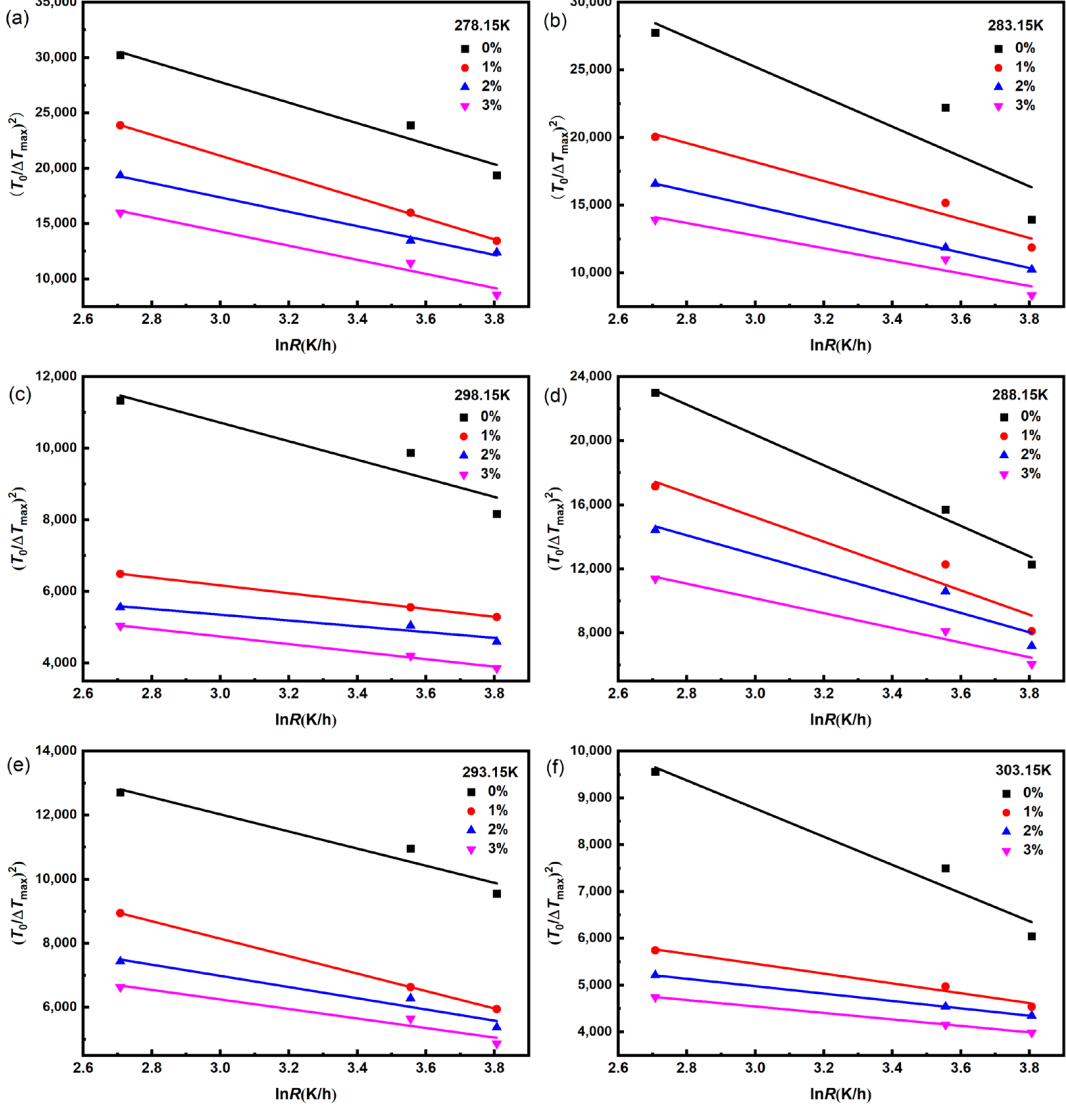
The fitting curves of (*T*_0_/Δ*T*_max_)^2^ and ln R of Na_2_SO_4_ at different dimethoxymethane concentrations by using Classical 3D nucleation theory model with different saturation temperatures: (**a**) 278.15 K; (**b**) 283.15 K; (**c**) 288.15 K; (**d**) 293.15 K; (**e**) 298.15 K; and (**f**) 303.15 K.

In order to investigate the effect of dimethoxymethane on the nucleation mechanism of Na_2_SO_4_, the classical 3D nucleation theory model was employed to fit the metastable zone width data of Na_2_SO_4_, as shown in Figure 3. The calculated results of the solid–liquid interface energy *γ* and pre-exponential factor *A* of Na_2_SO_4_ under different conditions are shown in Tables S3 and S4. Increases in saturation temperature and dimethoxymethane concentration increased the solid–liquid interface energy *γ*, which made crystal nucleation more difficult and led to experimental broadening of the metastable zone with an increase in the saturation temperature and dimethoxymethane concentration.

#### 2.1.3. The Induction Period of Na_2_SO_4_ Solution

**Figure 4:**
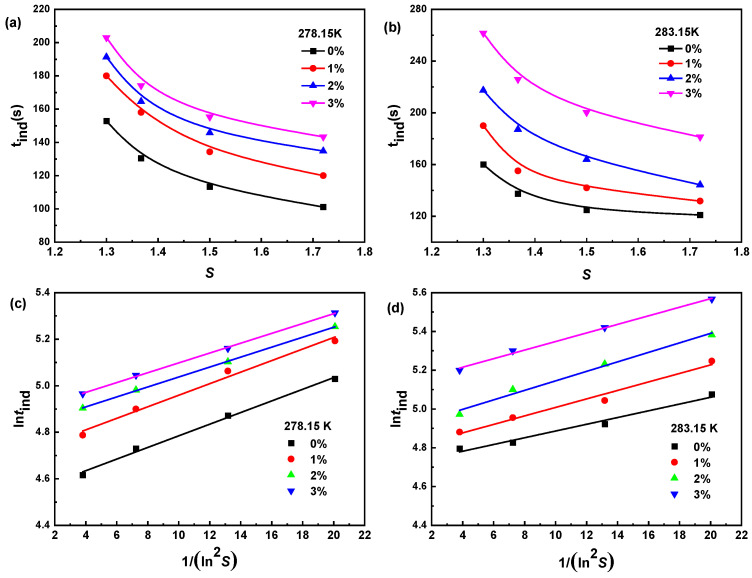
Effect of dimethoxymethane concentration and supersaturation on induction period of Na_2_SO_4_ and the fitting results of induction period data based on classical nucleation theory at different saturation temperatures: (**a**,**c**) 278.15 K and (**b**,**d**) 283.15 K.

The induction period is mainly controlled by the experimental environment where the material is located [29,30]. The effects of the saturation temperature, dimethoxymethane concentration, and supersaturation on the induction period of Na_2_SO_4_ are shown in Figure 4. The induction period of Na_2_SO_4_ increased with decreasing supersaturation, an increasing saturation temperature, and an increasing dimethoxymethane concentration, making crystal nucleation difficult, which was similar to the trend of the previously measured metastable zone width.

The classical nucleation theory was used to fit the induction period data, as shown in Figure 4. The solid–liquid interface energy (*γ*), critical nucleus size (r*), and critical Gibbs free energy ΔG* of the Na_2_SO_4_ crystallization system with different concentrations of dimethoxymethane and different supersaturations were calculated, as shown in Table 1. The *γ* decreased with increasing supersaturation, increased with an increasing dimethoxymethane concentration, and increased with an increasing temperature. The variation trends of r* and ΔG* were similar to those of *γ*. The above results indicate that crystal nucleation became difficult with decreasing solution supersaturation, an increasing dimethoxymethane concentration, and an increasing temperature. This theoretically explains the experimental phenomenon of the induction period data, which was consistent with the conclusion of the metastable zone.

In summary, the results of the analysis of the metastable zone width and induction period data indicated an effect on the nucleation mechanism of Na_2_SO_4_. The increase in dimethoxymethane concentration increased the solid–liquid interfacial energy and critical nucleation size, making crystal nucleation difficult.

### 2.2. Phase Equilibrium of Pseudo-Ternary Na_2_SO_4_-NaCl-H_2_O System in Simulated High-Salinity Wastewater

**Figure 5:**
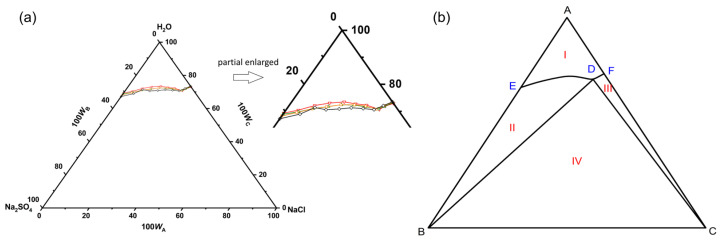
(**a**) The equilibrium phase diagrams and (**b**) schematic diagram of pseudo-ternary Na_2_SO_4_-NaCl-H_2_O system in simulated wastewater at 303.15 K (black line), 313.15 K (orange line), 323.15K (green line) and 333.15 K (red line). *W_a_*, *W_b_*, and *W_c_* are the mass fractions of NaCl, Na_2_SO_4_, and H_2_O, respectively. Points: A, H_2_O; B, Na_2_SO_4_; C, NaCl; D, the co-saturated point of Na_2_SO_4_ and NaCl; E and F, the saturated points of Na_2_SO_4_ and NaCl in the solution, respectively. Curves: DE and DF, the solubility curves of Na_2_SO_4_ and NaCl, respectively. Regions: AEDF (I), unsaturated solution; BDE (II), crystalline regions of Na_2_SO_4_; CDF (III), crystalline regions of NaCl; BDC (IV), crystalline regions of Na_2_SO_4_ and NaCl.

In the temperature range of 293.15 to 333.15 K, the equilibrium composition and solution density of the pseudo-ternary Na_2_SO_4_-NaCl-H_2_O system in simulated wastewater were determined, as listed in Table S5. The equilibrium phase diagrams of the pseudo-ternary system are shown in Figure 5. The composition of the simulated wastewater is shown in Table 2. In the temperature range of 303.15 to 333.15 K, the system had a co-saturation point, two solubility curves, an unsaturated zone, and three crystallization zones. The crystallization zone of Na_2_SO_4_ was larger than that of NaCl. The dissolution trends of NaCl and Na_2_SO_4_ in the simulated wastewater were the same as those in pure water. The solubility of Na_2_SO_4_ decreased with the addition of NaCl, which might have been due to the co-ionization effect. In addition, compared with the binary saturated solution, the decrease in the solubility of Na_2_SO_4_ at the ternary co-saturation point was much larger than that of NaCl, indicating that NaCl had a stronger salting out effect on Na_2_SO_4_ in the simulated wastewater system.

As shown in Figure S1, in the temperature range of 303.15 to 333.15 K, the co-saturation points position of Na_2_SO_4_ and NaCl shifted to the lower right as the temperature increased, indicating that the crystallization zone of Na_2_SO_4_ became larger while that of NaCl became smaller. In addition, the shift of the co-saturation point demonstrated that the salting out strength of NaCl to Na_2_SO_4_ became stronger with an increasing temperature, while the salting out effect of Na_2_SO_4_ to NaCl weakened. Therefore, in the salt recovery process of high-salinity wastewater from the coal chemical industry, NaCl could be added to the wastewater at a higher temperature until it reaches saturation, followed by being concentrated to reduce the water content and precipitate a large amount of Na_2_SO_4_ crystals, with the co-saturated wastewater being treated.

**Figure 6:**
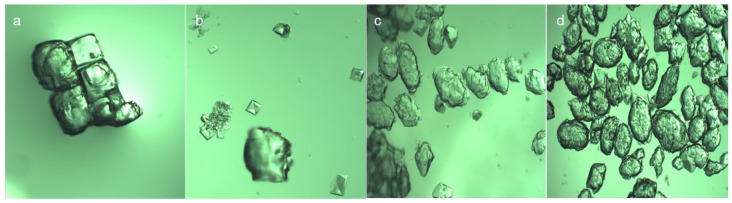
NaCl and Na_2_SO_4_ crystals observed under metallographic digital microscope. (**a**,**b**) are NaCl crystals and (**c**,**d**) are Na_2_SO_4_ crystals.

The morphologies of the NaCl crystals and Na_2_SO_4_ crystals were observed in the simulated wastewater, as shown in Figure 6. The NaCl crystal was a cube with fork depression, gradually transforming from a cube to an ellipsoid with a layered and relatively flat surface. As a contrast, the Na_2_SO_4_ crystal morphology was linear ellipsoid with a rough and uneven crystal surface and no obvious hierarchy. Under the simulated wastewater conditions, the addition of NaCl had a strong salting out effect on the saturated solution of Na_2_SO_4_ and the solubility of Na_2_SO_4_ decreased significantly, resulting in the nucleation rate of Na_2_SO_4_ crystal precipitation being greater than the growth rate, with a damaged crystal morphology.

### 2.3. Crystallization Kinetics of Na_2_SO_4_ in Simulated High-Salinity Wastewater

The results of the particle size analysis of the Na_2_SO_4_ crystals indicated that the crystal growth was independent of particle size. The kinetic equation of the Na_2_SO_4_ crystals in the simulated wastewater solution was obtained by an intermittent dynamic method, as shown in Table 3.

According to the nucleation rate model in Equation (13) and growth rate model in Equation (14), the 1stOpt software 5.0 was used to fit the kinetic experimental data. The crystal nucleation rate and growth rate equations of the kinetic model in simulated high-salinity wastewater were obtained as shown below.

(1) $$B^{0}=2.87712\times 10^{-15}\exp(\frac{2.69109\times 10^{4}}{RT})\Delta C^{1.91}M_{T}^{2.38}$$

where *B*^0^ is the nucleation rate, n·m^−3^·s^−1^; *M_T_* is the suspension density, kg·m^−3^; Δ*C* is the supersaturation, mol·L^−1^; *R* is the gas constant, J·K·mol^−1^; and *T* is the temperature, K.

(2) $$G=1.28\times 10^{-3}\exp(\frac{-2.20652\times 10^{4}}{RT})\Delta C^{-0.64}$$

where *G* refers to the growth rate, m·s^−1^; *T* is the temperature, K; *R* is the gas constant, J·K·mol^−1^; and Δ*C* is the supersaturation, mol·L^−1^.

According to the nucleation kinetics equation, the index of supersaturation was 1.91, which is less than 10. Therefore, the Na_2_SO_4_ crystallization was a secondary nucleation process. The growth kinetic equation showed that the supersaturation index was −0.64, which is less than 1, indicating that the growth of Na_2_SO_4_ crystals was controlled by surface reactions [31].

Both the growth rate and nucleation rate of Na_2_SO_4_ increased with an increasing temperature, especially the growth rate. The influence of supersaturation on the nucleation rate of Na_2_SO_4_ was greater than that on the growth rate, and the nucleation of Na_2_SO_4_ crystals was more likely to occur [32]. The nucleation rate of Na_2_SO_4_ was squared to the suspension density, i.e., the nucleation rate increased with an increase in the suspension density of Na_2_SO_4_. The crystallization rate of sodium salt in high-salt wastewater directly affects the desalting efficiency, and the grain size of sodium salt crystals also affects the selection of separation equipment and the setting of parameters [33]. Therefore, it is necessary to carry out research on crystallization kinetics and the identification of influencing factors to provide a basis for equipment selection and the optimization of process parameters.

## 3. Materials and Methods

### 3.1. Reagents

The main chemical reagents, including NaCl, Na_2_SO_4_, dimethoxymethane, silver nitrate, potassium chromate, barium chloride, hydrochloric acid, and absolute ethyl alcohol, etc., were used as-received from various chemical suppliers and were all analytically pure. All solutions were prepared with Milli-Q water.

### 3.2. Experimental and Analysis Methods

Methods for water quality analysis of coal chemical wastewater. A qualitative analysis of the total composition was performed to understand the unknown components and composition of the Ningxia coal chemical wastewater. Common inorganic salt ions, including SO_4_^2−^, Cl^−^, NO_3_^−^, Na^+^, Mg^2+^, and Ca^2+^, etc., were detected by ion chromatography. The total component analysis of organic compounds, including volatile and semi-volatile organic compounds, was performed by gas chromatography–mass spectrometry (GC-MS) after different pretreat methods, such as extraction, drying, concentration, or dilution, etc.

Solubility determination. In this study, the solubility was determined by the static equilibrium method [34]. Different concentrations of dimethoxymethane solutions were prepared, with excessive solute added. After being incubated in a programmed constant temperature and humidity incubator for 8 h and left to stand for 1 h, the supernatant was quickly filtered with a 0.45 μm filter membrane to determine the contents of SO_4_^2−^ and Cl^−^, and the solute content was analyzed and calculated. Each experimental point was repeatedly analyzed 3 times, and the average value was taken as the final experimental value. The SO_4_^2−^ contents were measured by the gravimetric method with excess barium chloride solution, while the Cl^−^ contents were quantified by the argentometric method with standard silver nitrate solution in the presence of potassium chromate (see the Supplementary Materials for the details), according to the national standard of China (GB/T 13025.8-2012 [35] and GB/T 13025.5-2012 [36]), respectively.

Determination of metastable zone. The laser method was applied [29]. According to the measured solubility data, the saturated solution of Na_2_SO_4_ was accurately prepared, added to the glass jacketed crystallizer, and connected to the laser monitoring system. The stirring rate was set as constant, and the low-temperature thermostat was controlled to cool down at a certain cooling rate. When the crystal was observed, the temperature was recorded at this time, and the metastable zone was obtained by combining with the solubility curve. The experiment was repeated 3~5 times.

Determination of induction period [37]. The measurement method of the induction period was the same as that of the metastable zone, which was also determined by the laser analysis method. After connecting the laser monitoring system, the solution was kept at a constant temperature for half an hour, and the readings of the thermometer and the digital laser power were recorded until they tended to be stable. The stopwatch was turned on when the solution reached the corresponding supersaturation and the changes in the digital laser power device were monitored until the turbidity phenomenon occurred. The experiment was repeated 3~5 times.

Determination of phase equilibrium data. With the saturated solution of one salt as the initial solution, a series of pseudo-ternary system solutions were prepared by adding another new salt with gradient concentrations [38]. The simulated wastewater solution with different ratios was placed in a constant-temperature oscillation box, oscillating for 9 h and standing for 1 h. The supernatant was then taken and filtered for the measurements of SO_4_^2−^, Cl^−^, and the liquid density. The solid was rapidly filtered, and the morphology of the crystal was observed by a metallographic digital microscope.

Determination of crystallization kinetics data. The intermittent dynamic method was used [39]. The simulated wastewater solution was prepared with saturated Na_2_SO_4_ added. The temperature was set according to the undercooling data, and the time of crystal nucleus appearance was judged by temperature changes and visual observation of whether there was crystal precipitation in the solution. With a large number of crystals precipitated, samples were taken, filtered, and dried every 5 min for analysis. The suspension density, solution supersaturation, and crystal particle size distribution were measured, and the sampling volume and sampling time were recorded at the same time.

### 3.3. Theoretical Models

#### 3.3.1. Thermodynamic Models for Solubility [40]

1. The Apelblat model

The Apelblat model was obtained on the basis of the Keck equation. The expression is as follows:

(3) $$lnx=A+\frac{B}{T}+ClnT$$

where *x* is the molar fraction of the solute; *T* is the absolute temperature, K; and *A*, *B*, and *C* represent different parameters to be estimated.

2. The Van ‘t Hoff model

The Van ‘t Hoff model was originally used to calculate the reaction equilibrium constant, and its expression is as follows:

(4) lnx=a+b/T

where *x* is the molar fraction of the solute; *T* is the absolute temperature, K; and *a* and *b* represent different parameters to be estimated.

3. The polynomial model

The expression of the polynomial model is as follows:

(5) $$x=A+B\times T+C\times T^{2}$$

where *x* is the molar fraction of the solute; *T* is the absolute temperature, K; and *A*, *B*, and *C* represent different parameters to be estimated.

#### 3.3.2. Models for Metastable Zone Width

Based on the Nyvlt’s equation [41] and the self-consistent Nyvlt-like model [42], the relationship between the width of the metastable zone and the cooling rate under the condition of a constant stirring rate is shown as follows:

(6) $$\ln\frac{\Delta T_{\max}}{T_{0}}=\frac{1-m}{m}\ln(\frac{\Delta H_{S}}{R_{G}T_{\lim}})+\frac{1}{m}\ln(\frac{f}{KT_{0}})+\frac{1}{m}\ln R$$

where Δ*T*_max_ is the width of the metastable zone; *m* is the nucleation order; *R_G_* and Δ*H_s_* are the ideal gas constant and dissolution enthalpy, respectively; *T*_lim_ represents the nucleation temperature; *f* is the number of entities per unit volume; *K* is the nucleation constant; and *R* is the cooling rate [43].

Classical nucleation theory [44] is widely used to fit metastable region data, and the expression is as follows:

(7) $$J=A\exp(\frac{-16\pi \gamma^{3}V_{S}}{3k_{B}^{3}T_{\lim}^{3}}\frac{1}{\ln^{2}S})$$

where *J* is the nucleation rate; *A* is the pre-exponential factor; *γ* is the solid–liquid interface energy; *V_s_* represents the volume of solute molecules; *T*_lim_ represents the nucleation temperature; and *S* represents supersaturation.

Combining Equations (6) and (7) and taking the logarithm, the following equations can be obtained.

(8) $${\left(\frac{T_{0}}{\Delta T_{\max}}\right)}^{2}=F_{1}(X+\ln T_{0}-\ln R)=F-F_{1}\ln R$$

(9) $$F_{1}=\frac{3}{16\pi }\frac{k_{B}^{3}T_{\lim}^{3}}{\gamma^{3}V_{S}^{2}}{\left(\frac{\Delta H_{S}}{R_{G}}\right)}^{2}$$

(10) $$X=\ln(\frac{A}{f}\frac{R_{G}T_{\lim}}{\Delta H_{S}})$$

where *A* refers to the pre-factor; *γ* is the solid–liquid interfacial energy; *V_s_* stands for the solute molecular volume; *T*_lim_ stands for the nucleation temperature; and *S* stands for the supersaturation. The solid–liquid interface energy *γ* and the pre-exponential factor *A* can be obtained from the slope and intercept.

#### 3.3.3. Models for Induction Period

Based on classical nucleation theory and the Gibbs–Thompson equation [44], the following equation can be obtained. The interface energy can be expressed by Equation (12), where *α* is the slope of Equation (11).

(11) $$\ln t_{\mathrm{in}d}=\gamma +\frac{16\pi \gamma^{3}V_{S}^{2}}{3k_{B}^{3}T_{\lim}^{3}\ln^{2}S}$$

(12) $$\gamma ={\left(\frac{3\alpha k_{B}^{3}T_{\lim}^{3}}{16\pi V_{S}^{2}}\right)}^{\frac{1}{3}}$$

where *t*_in*d*_ is the induction time; *γ* is the solid–liquid interfacial energy; *V_s_* stands for the solute molecular volume; *T*_lim_ stands for the nucleation temperature; and *S* stands for the supersaturation.

#### 3.3.4. Nucleation Kinetics Models [45]

The secondary nucleation of crystals is a common phenomenon in production and life, and the following empirical equation is usually used:

(13) $$B=K_{b}\exp(\frac{-E_{B}}{RT})M_{T}^{c}\Delta C^{d}$$

where *B* is the nucleation rate, n·m^−3^·s^−1^; *M_T_* is the suspension density, kg·m^−3^; Δ*C* is the supersaturation, mol·L^−1^; *R* is the gas constant, J·K·mol^−1^; *T* is the temperature, K; *c* and *d* are kinetic equation parameters; *K_b_* is the nucleation rate constant; and *E_B_* is the nucleation activation energy, J·mol^−1^.

In the whole range of the particle size distribution, the relationship between the grain size of the Na_2_SO_4_ crystals and the logarithm of the particle number density is approximately linear, so the growth of Na_2_SO_4_ crystals complies with the law of ΔL, showing a grain-size-independent growth law. Industrial production often adopts the following empirical equation:

(14) $$G=K_{g}\exp\left(\frac{-E_{a}}{RT}\right)\Delta C^{g}$$

where *G* refers to the growth rate, m·s^−1^; *K_g_* is the growth rate constant; *T* is the temperature, K; *R* is the gas constant, J·K·mol^−1^; *E*_a_ is the growth activation energy, J·mol^−1^; and Δ*C* is the supersaturation, mol·L^−1^.

## 4. Conclusions

(1) The effects of temperature and organic impurities on the solubility of Na_2_SO_4_ and NaCl were investigated. The solubility of Na_2_SO_4_ and NaCl increased with a rising temperature. Under certain temperature conditions, the solubility of Na_2_SO_4_ decreased with an increasing dimethoxymethane content in the solution.
(2) The metastable zone width and induction period of Na_2_SO_4_ increased with an increasing dimethoxymethane content, saturation temperature, and cooling rate. The theoretical reasons for this could be that the increase in the saturation temperature and dimethoxymethane content increased the solid–liquid interface energy and the critical crystal nucleation size, which was unfavorable for the nucleation of Na_2_SO_4_.
(3) The phase diagrams of the NaCl-Na_2_SO_4_-H_2_O pseudo-ternary system in the simulated wastewater were plotted in the temperature range of 303.15 to 333.15 K. Under the simulated wastewater conditions, the crystallization zone of Na_2_SO_4_ was larger than that of NaCl, and the density of the system was positively correlated with the amount of Na_2_SO_4_. The nucleation rate of Na_2_SO_4_ was greater than the growth rate due to the salting out effect.
(4) The crystallization kinetics equations of Na_2_SO_4_ in the simulated wastewater solution were obtained through kinetic experiments. The crystallization nucleation of Na_2_SO_4_ was a secondary nucleation process, controlled by surface reactions. A higher solution temperature and suspension density would be favorable for the crystallization and nucleation of Na_2_SO_4_.

## Figures and Tables

**Table 1:** The solid–liquid interfacial energy *γ* and critical nucleus size r* of Na_2_SO_4_ in water at different temperatures, supersaturations, and dimethoxymethane concentrations.

		*w* (Dimethoxymethane) (%)							
T/K	S	0		1		2		3	
		*γ*	r*	*γ*	r*	*γ*	r*	*γ*	r*
278.15	1.67	5.17	2.97	5.19	3.16	5.21	3.25	5.23	3.32
	1.45	5.19	3.34	5.21	3.52	5.24	3.60	5.26	3.68
	1.32	5.20	3.62	5.23	3.81	5.25	3.89	5.28	3.95
	1.25	5.21	4.03	5.23	4.21	5.26	4.28	5.29	4.36
283.15	1.67	7.21	3.36	7.23	3.54	7.25	3.62	7.27	3.71
	1.45	7.23	3.72	7.24	3.91	7.26	4.01	7.29	4.11
	1.32	7.24	4.01	7.25	4.20	7.27	4.28	7.30	4.35
	1.25	7.26	4.45	7.27	4.63	7.28	4.71	7.31	4.81

**Table 2:** Composition of simulated wastewater system.

Impurities	Concentrations/mg·L^−1^
sodium nitrate	100~300
magnesium sulfate	200~500
calcium chloride	200~500
diethyl ether	350~450
dimethoxymethane	1050~1150
1,1 dichloroethane	100~200
dibromomethane	150

**Table 3:** Crystallization kinetics data of Na_2_SO_4_ at different temperatures.

Test Number	Temperature (°C)	Supersaturation (mol·L^−1^)	Suspension Density (kg·m^−3^)	Crystallization Period (s)	Nucleation Rate (n·m^−3^·s^−1^)	Growth Rate (m·s^−1^)
1	10	0.39	20.18	300	8.11 × 10^−8^	3.45 × 10^−7^
2	10	0.36	23.20	600	3.28 × 10^−8^	1.50 × 10^−7^
3	10	0.33	25.68	900	6.14 × 10^−8^	1.76 × 10^−7^
4	10	0.28	27.98	1200	1.08 × 10^−7^	2.47 × 10^−7^
5	10	0.26	30.56	1500	8.90 × 10^−8^	4.77 × 10^−7^
6	15	0.37	24.10	300	1.59 × 10^−8^	7.07 × 10^−8^
7	15	0.29	27.58	600	6.33 × 10^−8^	2.08 × 10^−7^
8	15	0.19	30.70	900	5.11 × 10^−8^	2.48 × 10^−7^
9	15	0.17	33.56	1200	1.35 × 10^−7^	5.14 × 10^−7^
10	15	0.09	36.90	1500	1.12 × 10^−7^	5.17 × 10^−7^
11	20	0.49	68.40	300	1.09 × 10^−6^	2.35 × 10^−7^
12	20	0.26	90.62	600	7.28 × 10^−7^	2.35 × 10^−7^
13	20	0.15	99.56	900	1.37 × 10^−7^	5.86 × 10^−7^
14	20	0.11	111.84	1200	1.94 × 10^−7^	9.22 × 10^−7^
15	20	0.08	116.78	1500	1.33 × 10^−7^	5.88 × 10^−7^

## Data Availability

Data are contained within the article and Supplementary Materials.

## Supplementary Materials

The following supporting information can be downloaded at: https://www.mdpi.com/article/10.3390/molecules29091928/s1, Table S1: Experimental and theoretical values of mole fraction of Na_2_SO_4_; Table S2: Van’t Hoff equation parameters of Na_2_SO_4_ at different concentrations of dimethoxymethane; Table S3: The *γ* for Na_2_SO_4_ in water at different temperatures and concentrations of dimethoxymethane; Table S4: The *A* for Na_2_SO_4_ in water at different temperatures and concentrations of dimethoxymethane; Table S5: Phase equilibrium solubility data of the pseudo-ternary system (NaCl-Na_2_SO_4_-H_2_O) in simulated wastewater; Figure S1: The co-saturated points of the pseudo-ternary system (NaCl-Na_2_SO_4_-H_2_O) of simulated wastewater at T = (303.15 to 333.15) K. (*W_A_*, *W_B_* and *W_C_* are the mass fractions of NaCl, Na_2_SO_4_ and H_2_O, respectively; Text S1: Determination of sulfate ion and chloride ion.
