# Supplementary material for: Influence of Organic Impurities on Fractional Crystallization of NaCl and Na2SO4 from High-Salinity Coal Chemical Wastewater: Thermodynamics and Nucleation Kinetics Analysis
Source: Molecules. 2024 Apr 23;29(9):1928. doi: 10.3390/molecules29091928 (PMC11085368; doi:10.3390/molecules29091928)
Supplement: Supplementary file 1 [file molecules-29-01928-s001.zip › molecules-2913192-supplementary.pdf]

Supporting Information

Influence of organic impurities on fractional crystallization of NaCl and Na_2SO_4 from high-salinity coal chemical wastewater: thermodynamics and nucleation kinetics analysis

Molecules

Bo Shen, Bo Zhao, Hai Du, Yongsheng Ren, Jianwei Tang, Yong Liu, Quanxian Hua and Baoming Wang*

*Corresponding author: Baoming Wang**

E-mail address: ziqiangdere@126.com.

Table S1. Experimental and theoretical values of mole fraction of Na_2SO_4

T /K	$X^{exp}/100^{-2}$	Apelblat model		Van't Hoff model		polynomial equation	
		X^{cal} /10^{-2}	RD /%	X^{cal} /10^{-2}	RD /%	X^{cal} /10^{-2}	RD /%
0% dimethoxymethane							
278.15	0.827	0.679	17.903	0.789	4.598	0.902	9.132
283.15	1.255	1.130	9.904	1.273	1.439	0.922	26.520
288.15	2.007	1.863	7.161	2.020	0.639	1.603	20.147
293.15	2.979	3.041	2.083	3.156	5.926	2.945	1.162
298.15	4.596	4.916	6.965	4.857	5.669	4.947	7.637
303.15	8.034	7.875	1.977	7.370	8.271	7.611	5.265
ARDP/%			7.666		4.424		11.644
RMSD/10^{-3}			1.777		3.007		3.118
1% dimethoxymethane							
278.15	0.829	0.572	31.059	0.757	8.664	1.073	29.370
283.15	1.226	0.989	19.297	1.222	0.293	0.929	24.240
288.15	1.786	1.692	5.247	1.941	8.655	1.528	14.456
293.15	2.764	2.862	3.515	3.033	9.697	2.870	3.805
298.15	4.432	4.786	8.002	4.669	5.345	4.954	11.793
303.15	8.103	7.922	2.232	7.086	12.550	7.782	3.959
ARDP/%			11.558		7.534		14.604
RMSD/10^{-3}			2.232		3.857		3.976
2% dimethoxymethane							
278.15	0.783	0.535	31.696	0.719	8.106	1.190	52.033
283.15	1.185	0.936	20.978	1.170	1.240	1.032	12.891
288.15	1.730	1.619	6.401	1.871	8.164	1.617	6.488
293.15	2.656	2.767	4.185	2.944	10.852	2.947	10.951
298.15	4.330	4.676	7.998	4.563	5.385	5.020	15.934
303.15	7.993	7.817	2.204	6.970	12.798	7.836	1.964
278.15	0.783	0.535	31.696	0.719	8.106	1.190	52.033
ARDP/%			12.243		7.757		16.710
RMSD/10^{-3}			2.230		3.902		4.589
3% dimethoxymethane							
278.15	0.747	0.602	19.446	0.670	10.341	0.913	22.141
283.15	1.105	1.021	7.652	1.103	0.213	0.698	36.872
288.15	1.609	1.712	6.378	1.785	10.907	1.254	22.094
293.15	2.534	2.840	12.062	2.840	12.082	2.580	1.814
298.15	4.274	4.664	9.122	4.451	4.147	4.678	9.451
303.15	7.960	7.583	4.732	6.872	13.665	7.546	5.199
ARDP/%			9.899		8.559		16.262
RMSD/10^{-3}			2.665		3.075		4.239

Table S2. Van’t Hoff equation parameters of Na_2SO_4 at different concentrations of dimethoxymethane

dimethoxymethane	0%	1%	2%	3%
ΔS_d(J·mol^{-1}·K^{-1})	185.04	184.84	187.92	193.07
ΔH_d(kJ·mol^{-1})	62.67	62.71	63.68	65.28
R^2	0.9942	0.9843	0.9844	0.9809

Table S3 The γ for Na_2SO_4 in water at different temperatures and concentrations of dimethoxymethane

(mJ·m^{-2})

w(dimethoxymethane) (%)	0	1	2	3
278.15 K	7.23	10.28	11.49	12.46
283.15 K	9.82	13.39	15.13	14.44
288.15 K	9.95	11.53	12.78	15.17
293.15 K	12.18	12.68	15.14	17.04
298.15 K	12.47	14.79	16.34	18.84
303.15 K	17.04	16.99	20.22	21.51

Table S4 The *A* for Na_2SO_4 in water at different temperatures and concentrations of dimethoxymethane

(×10^{30} m^{-3}·h^{-1})

w(dimethoxymethane)(%)	0	1	2	3
278.15 K	3.91	1.84	2.84	1.92
283.15 K	3.84	2.53	2.58	2.63
288.15 K	1.82	1.36	1.55	1.42
293.15 K	1.56	3.55	9.87	3.69
298.15 K	10.37	3.49	126.1	47.71
303.15 K	3.09	30.32	93.30	31.58

Table S5 Phase equilibrium solubility data of the pseudo-ternary system ($NaCl$-Na_2SO_4-H_2O) in simulated wastewater

T/K	No.	Composition in liquid phase		Solution density
		$100\omega_{NaCl}$	$100\omega_{Na2SO4}$	$\rho/g\cdot cm^{-3}$
333.15	1	27.09	0	1.236
	2	25.37	2.74	1.246
	3	24.47	4.85	1.259
	4	20.30	7.41	1.270
	5	17.17	10.06	1.250
	6	13.82	12.68	1.275
	7	10.22	16.73	1.285
	8	6.75	21.11	1.295
	9	3.29	26.26	1.297
	10	0	30.67	1.303
323.15	1	26.83	0	1.228
	2	25.21	2.98	1.265
	3	24.04	5.11	1.277
	4	20.26	8.03	1.280
	5	16.97	10.73	1.273
	6	13.63	13.95	1.276
	7	10.52	17.72	1.285
323.15	8	6.84	22.17	1.305
	9	3.57	26.63	1.309
	10	0	31.10	1.314
313.15	1	26.67	0	1.220
	2	24.95	2.94	1.215
	3	23.59	5.63	1.227
	4	20.26	7.84	1.256
	5	17.06	10.54	1.268
	6	13.54	14.04	1.259
	7	10.08	18.32	1.278
	8	6.62	22.46	1.299
	9	3.29	27.17	1.298
	10	0	31.77	1.318
303.15	1	26.50	0	1.205
	2	24.81	3.10	1.212
	3	23.11	6.27	1.232
	4	20.05	8.93	1.244
	5	16.76	11.93	1.251
	6	13.84	15.31	1.263
	7	10.48	18.92	1.275
	8	7.17	21.44	1.287
	9	4.46	26.59	1.314
	10	0	32.76	1.319

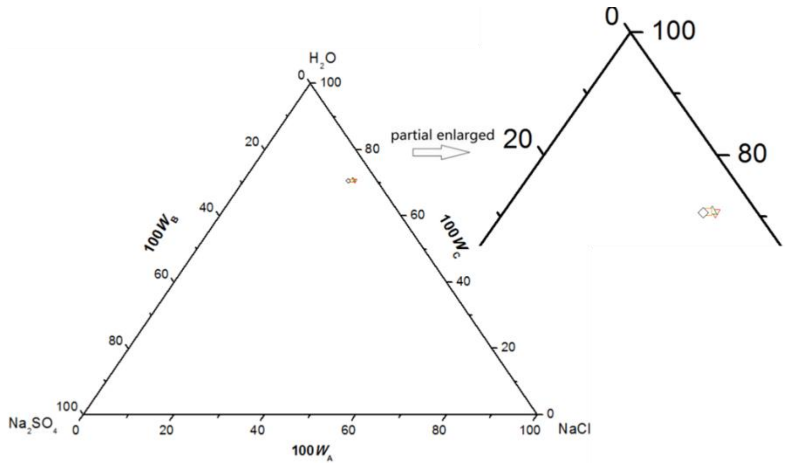

Figure S1 The co-saturated points of the pseudo-ternary system (NaCl-Na_2SO_4-H_2O) of simulated wastewater at T = 303.15 K (black point), 313.15 K (orange point), 323.15K (green point) and 333.15 K (red point). (W_A, W_B and W_C are the mass fractions of NaCl, Na_2SO_4 and H_2O, respectively).

Text S1

Determination of sulfate ion

The gravimetric method was applied to the quantification of sulfate ion based on the national standard of China (GB/T 13025.8-2012), which specifies methods for the determination of sulfate in salt products and salt chemical products. After being adjusted to acidity with hydrochloric acid, excessive barium chloride solution was added to the test sample to completely precipitate the sulfate ions. The barium sulfate precipitation was filtered, washed, dried and weighed. Weigh the precipitation every 30 minutes until the difference between two weightings is less than 0.0002g. The SO_4^{2-} content in the sample was calculated based on the precipitation mass with the following equation:

$$\omega = \frac{\left(m_1 - m_2\right) \times 0.4116}{m} \times 100\%$$

Where ω represents the mass fraction of sulfate ion; m_1 is the mass of barium sulfate and glass crucible, g; m_2 is the mass of glass crucible, g; 0.4116 is the conversion coefficient from barium sulfate to sulfate ion; m is the mass of the sample taken, g.

Determination of chloride ion

Similarly, the argentometric method was adopted for the determination of chloride ion according to the national standard of China (GB/T 13025.5-2012), which is suitable for the determination of chloride ion in salt products, salt chemical products and their raw materials. The sample solution was adjusted to neutral, titrated with silver nitrate standard titration solution, with potassium chromate as an indicator. When the solution appeared orange red without fading for 30 seconds, it was recorded as the titration end point. The content of chloride ion was calculated based on the volume of silver nitrate solution consumed with the following equation:

$$\omega = \frac{\left(V_1 - V_0\right) \times c\left(AgNO_3\right) \times 35.453}{m \times 1000} \times 100\%$$

Where ω represents the mass fraction of chloride ion; V_1 is the consumed volume of silver nitrate standard solution by the test sample, mL; V_0 is the consumed volume of silver nitrate standard solution in blank test, mL; $c(AgNO_3)$ is the concentration of silver nitrate standard solution, mol/L; 35.453 is the molar mass of chloride ion, g/mol; m is the mass of the sample taken, g; 1000 is the unit conversion coefficient.
